# Templated fabrication of hollow nanospheres with ‘windows’ of accurate size and tunable number

**DOI:** 10.1186/s11671-015-0795-5

**Published:** 2015-03-27

**Authors:** Duan Xie, Yidong Hou, Yarong Su, Fuhua Gao, Jinglei Du

**Affiliations:** School of Physical Science and Technology, Sichuan University, Chengdu, Sichuan 610064 China; Key Laboratory of High Energy Density Physics and Technology, Ministry of Education, Sichuan University, Chengdu, Sichuan 610064 China

**Keywords:** Nanosphere lithography technology, Temple-assisted deposition method, Hollow spheres

## Abstract

The ‘windows’ or ‘doors’ on the surface of a closed hollow structure can enable the exchange of material and information between the interior and exterior of one hollow sphere or between two hollow spheres, and this information or material exchange can also be controlled through altering the window’ size. Thus, it is very interesting and important to achieve the fabrication and adjustment of the ‘windows’ or ‘doors’ on the surface of a closed hollow structure. In this paper, we propose a new method based on the temple-assisted deposition method to achieve the fabrication of hollow spheres with windows of accurate size and number. Through precisely controlling of deposition parameters (i.e., deposition angle and number), hollow spheres with windows of total size from 0% to 50% and number from 1 to 6 have been successfully achieved. A geometrical model has been developed for the morphology simulation and size calculation of the windows, and the simulation results meet well with the experiment. This model will greatly improve the convenience and efficiency of this temple-assisted deposition method. In addition, these hollow spheres with desired windows also can be dispersed into liquid or arranged regularly on any desired substrate. These advantages will maximize their applications in many fields, such as drug transport and nano-research container.

## Background

Due to their unique properties, such as large internal void, doubled surface area, and low density, hollow spheres have attracted considerable interest in the past few decades, and many potential applications based on hollow spheres also have been demonstrated [1]. For instance, the gold or silicon hollow spheres have been demonstrated to own some special mechanical- or structure-dependent Mie scattering properties and can emit fascinating ‘music’-coherent acoustic vibrations or reveal a color nano-world in our bare eyes [2-4]. The large internal void has been used as carriers for the controlled encapsulation release of various substances such as drugs, bio-molecules, dyes, inks, and so on [5-8]. A perfect hollow sphere is indeed a hermetic space, a passageway, which will enable the exchange of substances or information between the interior and exterior spaces, and is a key element for maximizing its advantages (such as large internal void and doubled surface area) in the practical applications, and even for the formation of hollow spheres. In fact, most of the related applications are developed based on this passageway [5-17], which is usually formed through three ways: gaps between atoms or molecules (diffusion mechanism), gaps between nano-scale crystals, and apertures on the hollow sphere surface. However, these ways are usually random formed on the surface of hollow spheres and limit the precise control of the related substance or information exchange.

Template-assisted synthesis technology is the most popular method for the fabrication of hollow nano-structures, which usually needs PS spheres, PSA latex and metal particles as hard templates, or polymer micelles, gas bubbles, and surfactants (such as ionic organic surfactants, nonionic polymeric surfactants, and polymer surfactants) as soft templates in the fabrication process [17-22]. While temple-free method (Ostwald ripening process) is another newly-developed method, which can avoid the inherent disadvantages of template-assisted synthesis method, such as the complicated removal process of hard temple and the fickle morphology of soft temple. Based on these methods, a great number of hollow structures have been achieved, such as hollow nanospheres, hollow polyhedrons, and hollow spheres with double-yolk egg [23-25]. Particularly, the hollow octahedral cages with tunable surface aperture (windows) have been synthesized, and the influence of surface aperture on the electrochemical property in lithium ion batteries is also demonstrated [13]. However, none of these reported methods can be used to achieve the precise control of the size and number of ‘windows’ on the surface of hollow spheres. In this paper, we propose a new method based on the template-assisted deposition method to achieve the fabrication of hollow spheres with desired ‘windows’. The size and number of windows on the sphere surface can be easily controlled through employing different deposition parameters. In addition, a geometrical model has been developed for the simulation of this hollow sphere.

## Methods

### Design principle of hollow nanospheres

**Figure 1:**
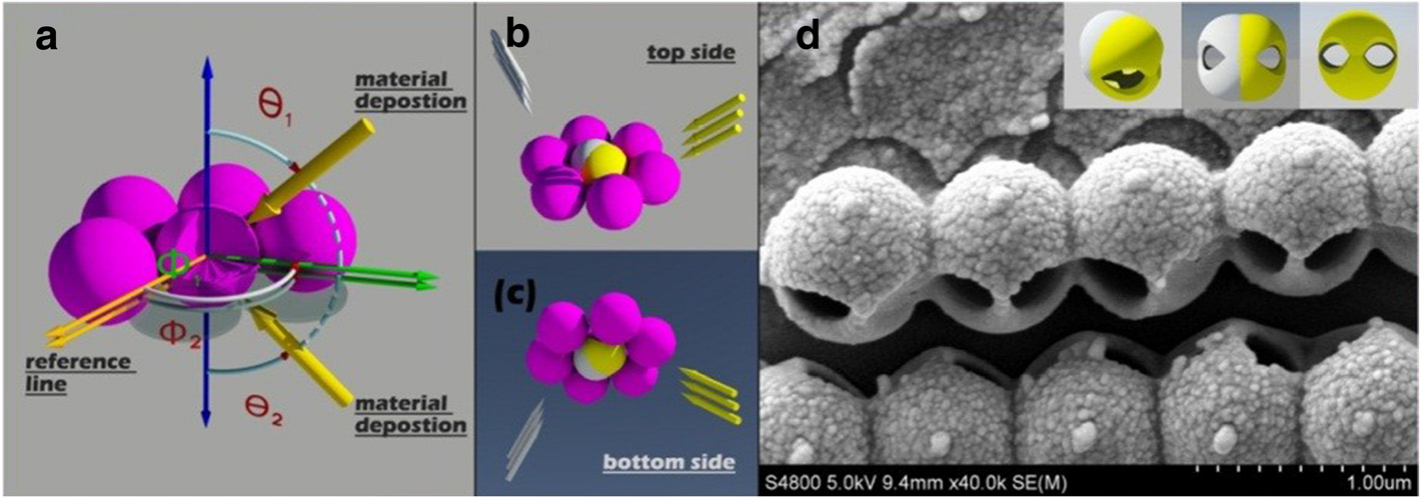
**Images of core-shell structures. (a)** Schematic of material vapor on a hexagonally close packed sphere monolayer; **(b)** material vapor from top side; **(c)** material vapor form bottom side. **(d)** The SEM image of the hollow spheres. In **(a)**, *θ*_1_, *θ*_2_ are the incident angle, and *φ*_1_, *φ*_2_ are the orientation angle; in **(d)**, the hollow spheres are fabricated with deposition parameters of *f*_top_ = *f*_bottom_ = 30[45°, 0°] + 30[45°, 180°], and the insets are the corresponding simulated images.

The nanosphere lithography is a kind of low-cost, material-independent, and simple technology, which can be used to fabricate 2-dimension particle array with the close packed spheres as mask and metallic vapor beam as irradiation source [26,27]. In the material deposition process, another kind of 3-dimension structures, i.e., shell-like structures, can also be formed on the spheres, and their number is very large due to the shadow effect [28,29]. This kind of structures can not only be used to pattern the sphere surface and form Janus or Patchy particles, but also be peeled off from the nanospheres to form a shell-like structure array [29-33]. In fact, these shell-like structures are formed through the material deposition from one side of the nanosphere mono-layer. When the PDMS stamp is applied to achieve the material deposition on the entire surface of the nanospheres, a new structure (core-shell structure) can be formed, as shown in Figure 1. In addition, these core-shell nanostructures can be further transformed to hollow nanospheres through a proper chemical treatment and lead to the formation of hollow spheres with specific windows on the surface.

Figure 1b and c is the schematic diagrams of the material deposition from the top and bottom sides of the closely packed nanosphere array monolayer with hexagonal symmetry, respectively. *θ*, *φ* are the key deposited parameters in the fabrication process, where *θ* is defined as the incidence angle of the material vapor beam measured from the substrate side and *φ* is the angle to describe the monolayer orientation: In the case of deposition from the top side, *φ*_1_ is defined as the anticlockwise rotating angle corresponding to the reference line; in the case of deposition from the bottom side, *φ*_2_ is defined as the clockwise rotating angle (as shown in Figure 1a). The insets in Figure 1d are the 3-dimensional images of the obtained hollow nanospheres through the material deposition on both sides of the nanosphere array monolayer. The deposition parameters are *f*_top_ = *f*_bottom_ = 30[45*°*, 0*°*] + 30[45*°*, 180*°*], which means that we employ twice the material deposition on each side and the deposition parameters are 30[45*°*, 0*°*] and 30[45*°*, 180*°*], respectively. Here, 30 nm is the deposition thickness defined as the thickness on the plane perpendicular to the vapor beam; 45° is the deposition angle, i.e., *θ* = 45*°*, 0°, and 180° are the monolayer orientation angles, i.e., *φ* = 0*°* or 180*°*. The simulated and SEM images of the hollow spheres are shown in Figure 1d, where we can see that the experiment results fit well with simulations.

### The fabrication process of hollow nanospheres

**Figure 2:**
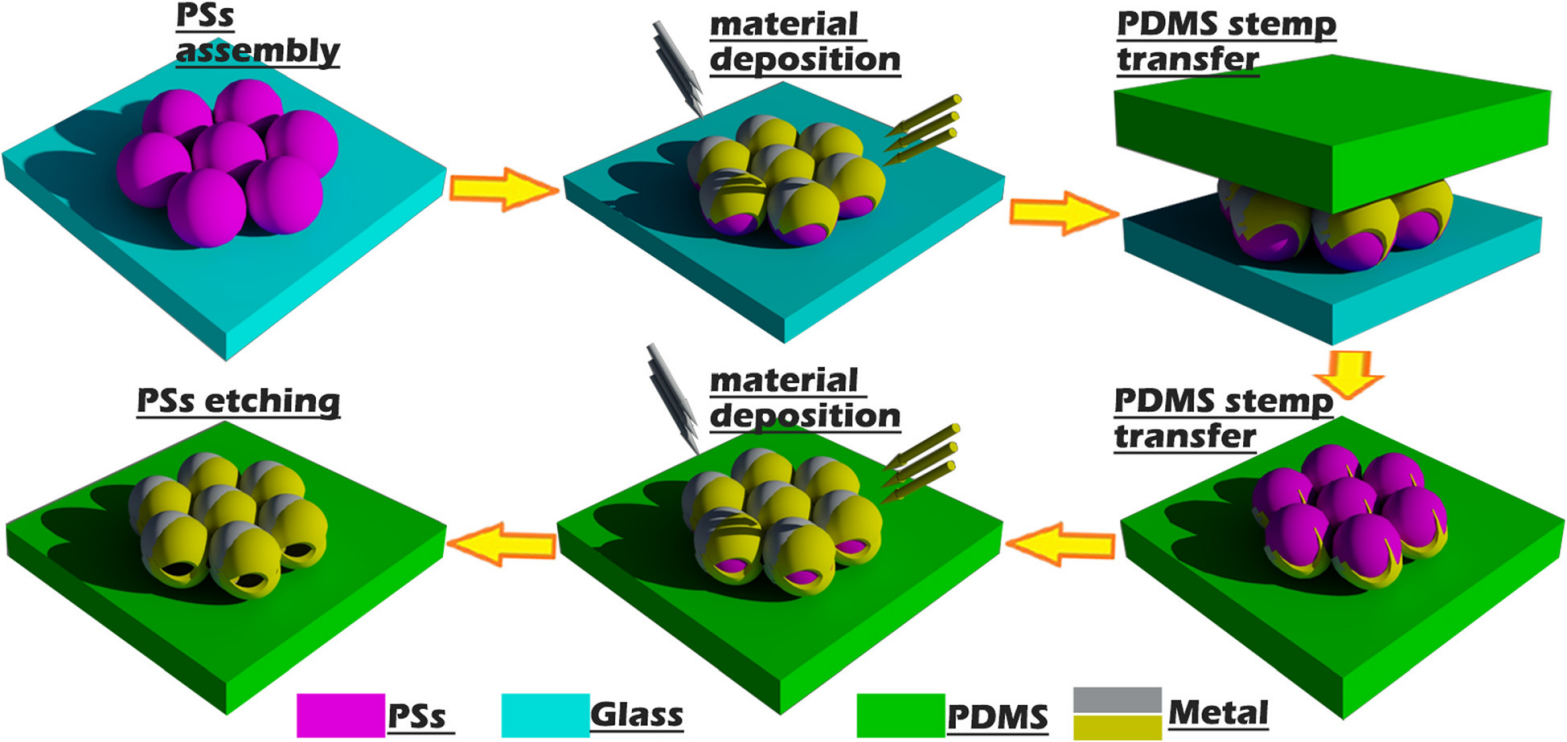
**Schematic of the fabricating process of hollow spheres.**

The detail fabrication process of hollow nanospheres is shown in Figure 2.

Firstly, a monolayer of PSs array with hexagonal symmetry is assembled on glass substrate via the method described in Ref. [33]. The monolayer orientation is confirmed by the scanning electron microscope (SEM), and a label line is made on a suitable position as reference line for the confirmation of the angle *φ* in the next step.

Secondly, the silver deposition on the closely packed PSs array monolayer from the top side is performed inside a vacuum thermal evaporation system. The base pressure, deposition rate, and temperature are 4 × 10^−4^ Pa, 0.1 nm/s, and 30°C, respectively.

Thirdly, a polydimethylsiloxane (PDMS) stamp is placed on the top surface of the silver-coated PSs array monolayer with a uniform pressure of about 500 g/cm^2^. After lifted up, this particle monolayer is transferred to the surface of the PDMS stamp due to the larger van der Walls force with the PDMS stamp compared with the silicon substrate. Especially, the reference line should also be properly marked on the PDMS stamp.

Fourthly, this PDMS stamp with the silver-coated PSs array monolayer on the surface is placed in the vacuum chamber to achieve the material deposition from the bottom side of the PS arrays monolayer, and the deposition condition is the same with that described in step 2.

Finally, the PDMS stamp together with the silver-coated PSs array monolayer is immersed in tetrahydrofuran for 3 min to etch away the PSs, and then this sample is dried in fume hood at room temperature. Six hours later, the hollow nanospheres are obtained on the PDMS stamp as shown in Figure 1d.

The PDMS stamp for inverting the PS array monolayer is produced by curing the elastomer and curing agent (10:1 *w/w*) at 80°C in an oven. To make the PDMS stamp without any bubble in it, the viscous mixture of PDMS is placed in a vacuum container with pressure of 4 Pa for the first 30 min and 4 × 10^−4^ Pa for the following 1 h. (The bubbles in the PDMS stamp can wrinkle the stamp surface at the pressure of 4 × 10^−4^ Pa, affect the PS array monolayer orientation, and thus the obtained hollow spheres.) In addition, the PDMS stamp is cut to the shape of the glass substrate, which is very useful for the mark of the reference line and the confirmation of the angle *φ*.

### The parameter analysis and experiment discussions

The material deposition from one side of the PS array monolayer will lead to the formation of a great number of shell-like structures due to the shadow effect from neighboring particles. And the deposition from two sides will lead to the addition of another parameter in the fabrication process of new structures, and thus the formation of more structures when compared with the deposition from one side. In this case, it becomes very important to choose suitable parameters to fabricate the hollow nanospheres with desired windows before we start the experiment process. Fortunately, all of the hollow spheres obtained through this method can be analyzed and simulated with the help of our established geometric model, and the size and number of the windows on the hollow sphere surface also can be calculated accurately. In the following discussion, we will choose some representative cases to discuss the influence of the key parameters on the obtained hollow nanospheres.

#### Influence of angle *θ*

**Figure 3:**
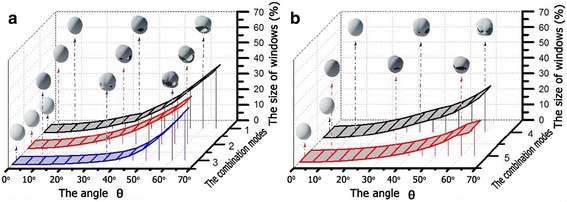
**The total size of windows on hollow sphere surface varied as a function of the angle** ***θ*** **. (a)** including modes 1, 2, 3 and **(b)** including modes 4, 5. The insets are the corresponding simulated images of the hollow spheres and the shell-like structure obtained through the deposition from the top side of PS array monolayer. The deposition parameters are *f*_top_ = *f*_bottom_ = 30[*θ*, 0°] + 30[*θ*, 180°] (mode 1), *f*_top_ = *f*_bottom_ = 30[*θ*, 30°] + 30[*θ*, 210°] (mode 2), *f*_top_ = *f*_bottom_ = 30[*θ*, 0°] + 30[*θ*, 120°] + 30[*θ*, 240°] (mode 3), *f*_top_ = *f*_bottom_ = 30[*θ*, 30°] + 30[*θ*, 150°] + 30[*θ*, 270°] (mode 4), and *f*_top_ = 70[0°, 0°]/*f*_bottom_ = 30[*θ*, 0°] + 30[*θ*, 180°] (mode 5), respectively.

The angle *θ* is one of the most important parameters determining the total size of windows on the hollow sphere surface. We choose some representative combination modes to reveal the influence of angle *θ*, as shown in Figure 3. Modes 1 and 2 represent the hollow spheres obtained through four-step material deposition, where the deposition parameters are *f*_top_ = *f*_bottom_ = 30[*θ*, 0*°*] + 30[*θ*, 180*°*] (for mode 1) and *f*_top_ = *f*_bottom_ = 30[*θ*, 30*°*] + 30[*θ*, 210*°*] (for mode 2). And a maximum window size of up to 35% can be obtained in mode 1. Modes 3 and 4 represent the hollow spheres obtained through six-step material deposition, where the deposition parameters are *f*_top_ = *f*_bottom_ = 30[*θ*, 0*°*] + 30[*θ*, 120*°*] + 30[*θ*, 240*°*] (for mode 3) and *f*_top_ = *f*_bottom_ = 30[*θ*, 30*°*] + 30[*θ*, 150*°*] + 30[*θ*, 270*°*] (for mode 4). And the maximum window size is about 35% in mode 3. Mode 5 represents a hollow sphere obtained through three-step material deposition, where the deposition parameters are *f*_top_ = 70[0*°*, 0*°*] ≠ *f*_bottom_ = 30[*θ*, 0*°*] + 30[*θ*, 180*°*]. And the maximum window size is about 20%.

In general, these curves show that the total sizes of windows all increase with the increase of angle *f*_top_ = *f*_bottom_ = 30[*θ*, 0*°*] + 30[*θ*, 180*°*], as shown in Figure 3. However, when the deposition angle *θ* is too large, the structures deposited from the top and bottom sides of the PS array monolayer will not have intersections with each other. And this will lead to the separation of the structures deposited from the top and bottom sides and damage the hollow spheres when the PS spheres have been etched away. Thus, we can get an upper limit *θ*_upper_, i.e., *θ* = *θ*_upper_, to obtain a completeness hollow sphere. The values of *θ*_upper_ are 66° for the modes 1 and 3 and 60° for the modes 2 and 4. For mode 5, the value of *θ*_upper_ can reach to 90° in theory. However, the intersections between the top and bottom structures should be firm enough to form a perfect hollow sphere when the PS sphere is etched away the experiment. Thus, the deposition angle *θ* and thickness *k* should be properly considered in the experiment.

In addition, the *θ* also can affect the number of windows on the obtained hollow sphere surface. When we choose θ = 35°, four windows can be achieved on the hollow sphere surface obtained through mode 1, as shown in Figure 3. And this window number is 6 for modes 2, 3, and 4, and 4 for mode 5. When θ = 60°, two windows are formed on the mode 1 and mode 5 hollow spheres, four for mode 2, three for mode 3, and six for mode 4. These simulated results indicate that we can effectively modify the window number on hollow sphere surface through using different deposition angle *θ*.

#### Influence of angle *φ*

**Figure 4:**
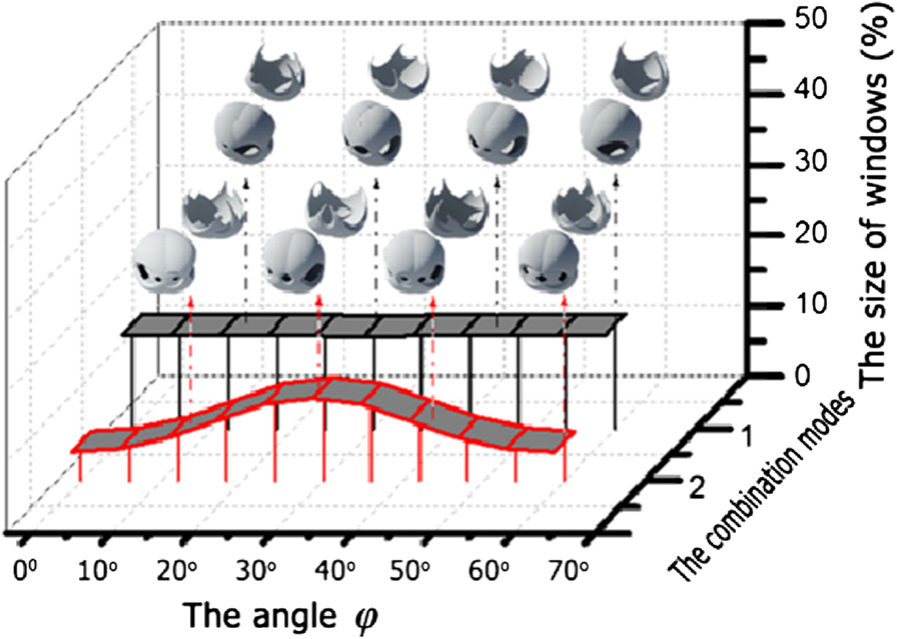
**The total size of windows on hollow sphere surface varied as a function of the angle** ***φ*** **.** The insets are the corresponding simulated images of the hollow spheres and the shell-like structure obtained through the deposition from the top side of PS array monolayer. The deposition parameters are *f*_*top*_ = *f*_*bottom*_ = 30[45°, *φ*] + 30[45°, *φ* + 180°](mode 1) and *f*_*top*_ = *f*_*bottom*_ = 30[45°, *φ*] + 30[45°, *φ* + 120°] + 30[45°, *φ* + 240°] (mode 2).

The angle *φ* is another key parameter in determining the total size of windows on the hollow sphere surface, representing the azimuth angle of PS array. Figure 4 shows the relationship between the total window size and the angle *φ*, where the deposition parameters for modes 1 and 2 are: *f*_top_ = *f*_bottom_ = 30 [45, *φ*] + 30[45, *φ* + 180°] and *f*_top_ = *f*_bottom_ = 30[45°, *φ*] + 30[45°, *φ* + 120°] + 30[45°, *φ* + 240°], respectively. For the closely packed PS array with hexagonal symmetry, the obtained hollow spheres with *φ* of different values have a period of *T*_*φ*_ = 60°. Thus, we just need to discuss the influence of *φ* with range from 0° to 60° on the obtained hollow spheres. The insets are the simulated hollow spheres with *φ* of the related values (bottom) and the related shell-like structures obtained through the material deposition from the top side (top). In fact, the shell-like structures with deposition parameters of *f*_top_ and *f*_bottom_ are mirror images of each other, when *f*_top_ = *f*_bottom_. And the hollow spheres (shown by the bottom insets in Figure 4) are the combination of the shell-like structures on the top and their mirror structures. The curves shown in Figure 4 indicate that the angle *φ* has a little influence on the total size of windows on the obtained hollow nanosphere surface, especially for mode 1. However, the shape of the windows on hollow sphere surface shown in Figure 4 has an obvious change as the angle *φ* increases, which indicates that the shape can be properly modified through the use of *φ* with different values. Thus, the shape, size, and number of windows, all can be properly optimized through the change of the values of the deposition parameter *θ* and *φ*.

#### Influence of other parameters

**Figure 5:**
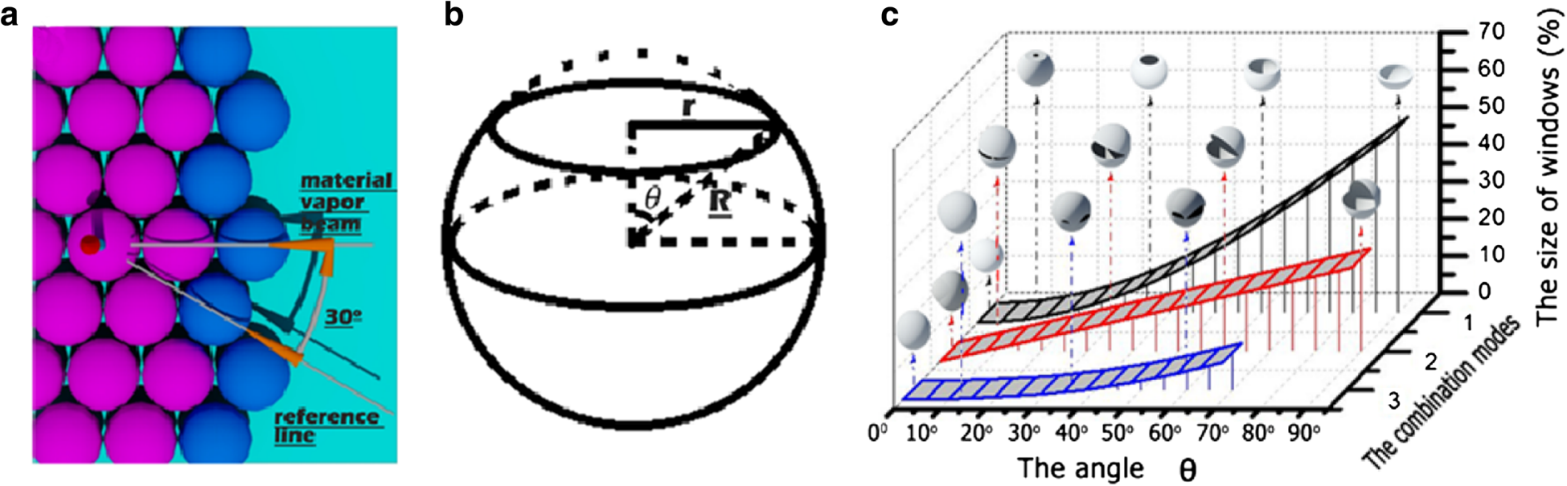
**The windows on hollow sphere surface. (a)** The material deposition schematic on the edge of PS array; **(b)** the schematic of spherical crown. *R*, *r*, and *θ* are the sphere radius, circular radius on the top surface of spherical crown, and the angle related to *r* and *R*, respectively. **(c)** The total window size on hollow sphere surface varied as a function of the angle *θ*. Mode 1 represents the hollow sphere obtained through reshaping the nanoshell, modes 2 and 3 represent the hollow sphere with *f*_top_ = 30[0°, 0°]/*f*_*bottom*_ = 30[*θ*, 0°] (mode 2) and *f*_top_ = 70[0°, 0°]/*f*_bottom_ = 30[80°, 30°] + 30[*θ*, 210°] (mode 3).

In addition to the angle *θ* and *φ*, the windows on hollow sphere surface also can be affected through some special cases. Figure 5a shows the material deposition schematic on the edge of PS array (the blue spheres), where 30° is the value of azimuthal angle of PS array, i.e., the angle of material vapor beam related to the reference line. To investigate the hollow spheres formed on the edge PS spheres, two kinds of deposition parameters are chosen, as shown in Figure 5c: one is mode 2, *f*_top_ = 30[0°, 0°]/*f*_bottom_ = 30[*θ*, 0°]; the other is mode 3, *f*_top_ = 70[0°, 0°]/*f*_bottom_ = 30[80°, 30°] + 30[*θ*, 210°]. The window size increases with the increase of angle *θ* in both modes, and a maximum window size of about 20% can be achieved in mode 2. The values of *θ*_upper_ for modes 2 and 3 are 90° and 70°, respectively. In theory, the window number is always 1 for mode 2, and 3 for mode 3 when *θ* < *θ*_upper_. Certainly, the hollow spheres obtained in combination with mode 2 (shown in Figure 5c) also can be achieved on the discrete PS array, where the distance between PS spheres is large enough that the shadow effect from the neighboring spheres can be eliminated. In the experiment, the discrete PS array and the edge of the close packed PS array can be obtained through the swelling and transferring technology of PDMS stamp, respectively, as described in Ref. [34].

For the fabrication of hollow spheres with one window, we also can apply oxygen ions to etch nanoshells, which are obtained through chemical reduction method, as described in Ref. [35]. As shown in Figure 5b, the window size can be described by the variate *θ*, r, and R, where *θ* = arcsin (*r*/*R*). The relation between the window size and variate *θ* is shown in Figure 5c, the combination mode 1, where we can get that a maximum window size of 50% can be achieved, and the values of *θ*_upper_ are 90°.

#### Experimental discussions

**Figure 6:**
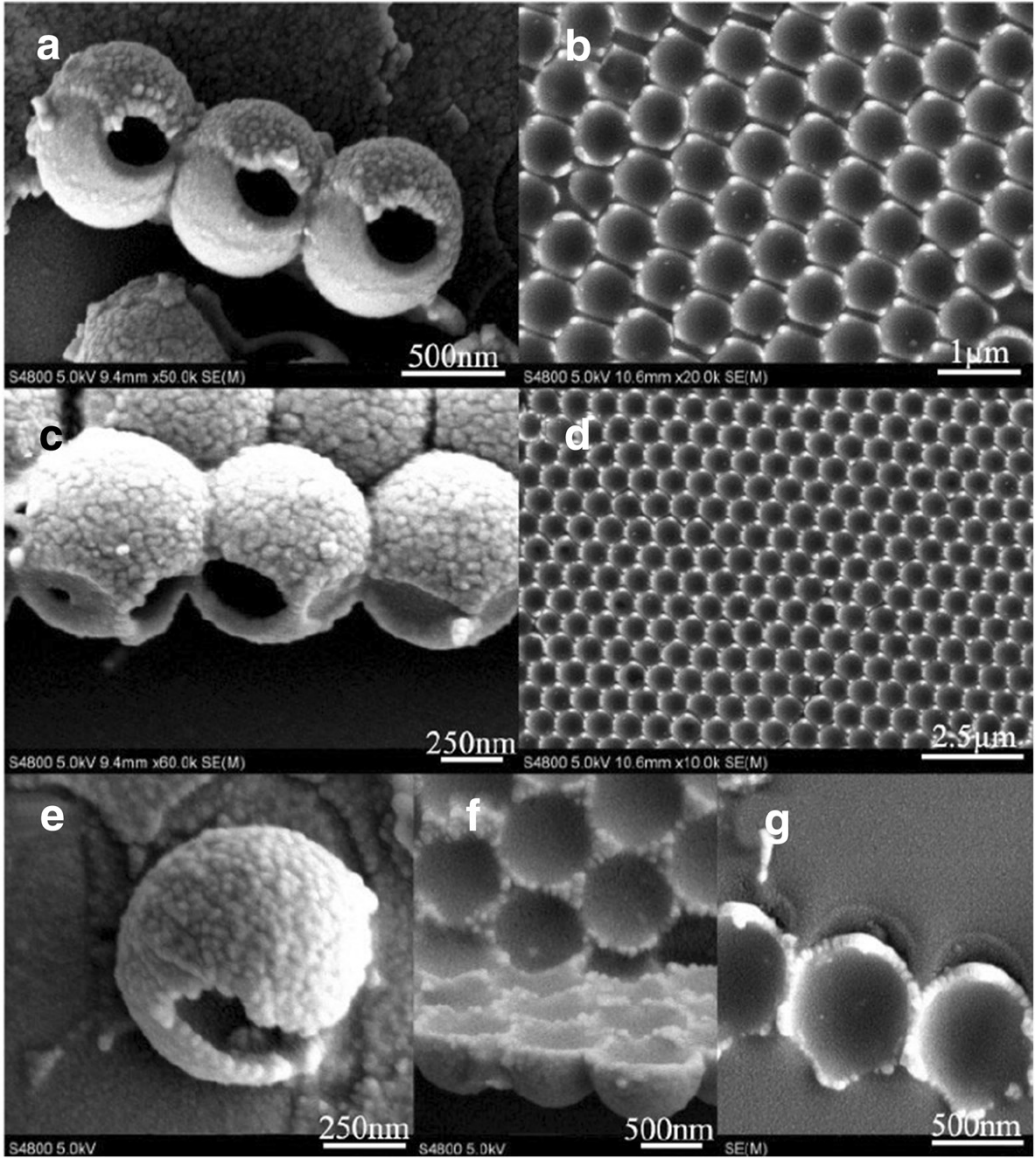
**The SEM images of fabricated structures. (a)** *f*_top_ = *f*_bottom_ = 50[45°, 30°] + 50[45°, 210°]; **(b)** *f*_top_ = 50[45°, 0°] + 50[45°, 180°]; **(c)** *f*_top_ = *f*_bottom_ = 50[45°, 0°] + 50[*θ*, 180°]; **(d)** *f*_top_ = 50[45°, 0°] + 50[45°, 180°]; **(e)** *f*_top_ = 70[0°, 0°]/*f*_bottom_ = 50[30°, 0°] + 50[30°, 180°]; **(f)** *f*_top_ = 70[0°, 0°]; **(g)** *f*_top_ = 50[30°, 0°] + 50[30°, 180°].

The fabrication method for the simulated hollow spheres is described in detail in the experimental section, and the fabricated results shown in Figure 1d indicate the validity of this method. The images in Figure 6 left, i.e., (a), (c), and (e), show some other hollow spheres, and the shell-like structures, which are used to construct these hollow spheres, are shown on the right. These structures all agree well with the simulation results, indicating the efficiency of the fabrication technology. Certainly, there are some differences between the simulated and fabricated structures, especially for the unsubstantial parts which can be destroyed by the surface tension and the gravity in the wet etching process. However, this difference can be reduced through the use of more deposition material or other methods, such as the optimization of processing conditions. Through these experiment results and analyses described above, we can obtain the hollow nanospheres with ‘windows’ of tunable size and number, and all of the hollow nanospheres can be designed with the help of our established geometric model before the experiment process.

The hollow spheres have many advantages as described in the introduction, and one of them is the large internal void, which can be used as carriers, nano-scale reactors, and so on. For the achievement of these applications, how to put the desired substances into the interior of the hollow spheres is a very important problem. Beside the method using windows as passageway, we also can employ another very convenient method to solve this problem for the hollow spheres obtained in this paper. Before the fabrication of the hollow nanospheres, the desired substances can be firstly deposited on the PS array, and then begin to fabricate the hollow spheres. After the PS spheres are etching away, these substances are left in the interior. When the deposited substances used here is very little, there is almost no effect on the obtained hollow spheres. In addition, this method described here also can be used to achieve the fabrication of core-shell structures or ‘Janus’ or ‘Patchy’ particles, when etching process is removed.

## Conclusion

In summary, hollow spheres with windows of accurate size and tunable number have been successfully fabricated through the proposed temple-assisted deposition method. Through the use of different PSs array and the control of the material deposition angle (i.e., *θ* and *φ*), hollow spheres with windows of total size from 0% to 50% and number from 1 to 6 are achieved. In theory, a geometrical model is established to achieve the simulation and design of these surface windows before the start of fabrication process, which will greatly reduce the groping time and cost in the experiment. These hollow spheres with different windows can be dispersed into liquid or arranged regularly on any desired substrate. And the substances can be put into the hollow sphere interior through their surface windows or the pre-deposition process. The fabricated hollow sphere with different windows will find potential applications in many fields, such as nano-carrier, drug transport, and nano-research container.

## References

[1] Hu J, Chen M, Fang X, Wu L (2011). Fabrication and application of inorganic hollow spheres. Chem Soc Rev.

[2] Retsch M, Schmelzeisen M, Butt HJ, Thomas EL (2011). Visible mie scattering in non absorbing hollow sphere powders. Nano Lett.

[3] Still T, Sainidou R, Retsch M, Jonas U, Spahn P, Hellmann GP (2008). The ‘Music’ of core-shell spheres and hollow capsules: influence of the architecture on the mechanical properties at the nanoscale. Nano Lett.

[4] Dowgiallo AM, Schwartzberg AM, Knappenberger KL (2011). Structure-dependent coherent acoustic vibrations of hollow gold nanospheres. Nano Lett.

[5] Ben T, Ren H, Ma S (2009). Targeted synthesis of a porous aromatic framework with high stability and exceptionally high surface area. Angew Chem.

[6] Zhu Y, Shi J, Shen W, Dong X, Feng J, Ruan M (2005). Stimuli-responsive controlled drug release from a hollow mesoporous silica sphere/polyelectrolyte multilayer core-shell structure. Angew Chem.

[7] Cochran JK, Curr O (1998). Ceramic hollow spheres and their applications Solid state mater. Sci.

[8] Huang H, Remsen EEJ (1999). Facile preparation of hollow hydroxyapatite microspheres. Am Chem Soc.

[9] Keng PY, Kim BY, Shim IB, Sahoo R, Veneman PE, Armastrong NR (2009). Colloidal polymerization of polymer-coated ferromagnetic cobalt nanoparticles into Pt-Co_3_O_4_ nanowires. ACS Nano.

[10] Koo B, Xiong H, Slater MD, Prakapenka VB, Balasubramanian M, Podsiadlo P (2012). Hollow iron oxide nanoparticles for application in lithium ion batteries. Nano Lett.

[11] Zhou JS, Song HH, Chen XH, Zhi LJ, Yang SY, Huo JP (2009). Oxidation conversion of carbon-encapsulated metal nanoparticles to hollow nanoparticles. Chem Mater.

[12] Li J, Zeng HC (2005). Size Tuning, functionalization, and reactivation of Au in TiO_2_ nanoreactors. Angew Chem.

[13] Wang X, Yu L, Wu XL, Yuan F, Guo YG, Ma Y (2009). Highly photocatalytic ZnO/In_2_O_3_ heteronanostructures synthesized by a coprecipitation method. Phys Chem C.

[14] Wang X, Wu XL, Guo YG, Zhong Y, Cao X, Ma Y (2010). Synthesis and lithium storage properties of Co_3_O_4_ nanosheet-assembled multishelled hollow spheres. Adv Funct Mater.

[15] Wang X, Zhong Y, Zhai T, Guo Y, Chen S, Ma Y (2011). One-dimensional inorganic nanostructures: synthesis, field-emission and photodetection. Mater Chem.

[16] Chang YE, Youn DY, Ankonina G, Yang DJ, Kim HG, Rothschild A (2009). Fabrication and gas sensing properties of hollow SnO_2_ hemispheres. Chem Commun.

[17] Shchukin DG, Caruso RA (2004). Template synthesis and photocatalytic properties of porous metal oxide spheres formed by nanoparticle infiltration. Chem Mater.

[18] Wang D, Song C, Hu Z, Fu XJ (2005). Fabrication of hollow spheres and thin films of nickel hydroxide and nickel oxide with hierarchical structures. Phys Chem B.

[19] Sun Y, Xia Y (2002). Shape-controlled synthesis of gold and silver nanoparticles. Science.

[20] Liu T, Xie Y, Chu B (2000). Use of block copolymer micelles on formation of hollow MoO_3_ nanospheres. Langmuir.

[21] Wang X, Li Y (2003). Fullerene-like rare-earth nanoparticles. Angew Chem Int Ed.

[22] Collins AM, Spickermann C, Mann S (2003). Synthesis of titania hollow microspheres using non-aqueous emulsions. J Mater Chem.

[23] Wang X, Yu L, Hu P, Yuan F (2007). Synthesis of single-crystalline hollow octahedral NiO. Cryst Growth Des.

[24] Lou XW, Yuan C, Archer LA (2007). Shell-by-shell synthesis of tin oxide hollow colloids with nanoarchitectured walls: cavity size tuning and functionalization. Small.

[25] Wang X, Liao M, Zhong Y, Zheng JY, Tian W, Zhai T (2012). ZnO hollow spheres with double-yolk egg structure for high-performance photocatalysts and photodetectors. Adv Mater.

[26] Guan BT, Xiang SK, Wang BQ, Sun ZP, Wang Y, Zhao KQ (2008). Direct benzylic alkylation via Ni-catalyzed selective benzylic sp3 C-O activation. Am Chem Soc.

[27] Zhang G, Wang D, Möhwald H (2007). Ordered binary arrays of Au nanoparticles derived from colloidal lithography. Nano Lett.

[28] Liu J, Maaroof AI, Wieczorek L, Cortie MB (2005). Fabrication of hollow metal ‘Nanocaps’ and their red-shifted optical absorption spectra. Adv Mater.

[29] Christopher Love J, Gates BD, Wolfe DB, Paul KE, Whitesides GM (2002). Fabrication and wetting properties of metallic half-shells with submicron diameters. Nano Lett.

[30] Nakanishi T, Michinobu T, Yoshida K (2008). Nanocarbon superhydrophobic surfaces created from fullerene-based hierarchical supramolecular assemblies. Adv Mater.

[31] Pawar AB, Kretzschmar I (2008). Patchy particles by glancing angle deposition. Langmuir.

[32] Liddell CM, Summers CJ, Gokhale AM (2003). Stereological estimation of the morphology distribution of ZnS clusters for photonic crystal applications. Mater Charact.

[33] Wen Z, Yuanyuan W, Chengyin W, Ming Z, Guanxiu D (2013). Fabrication of large-area 3-D ordered silver-coated colloidal crystals and macroporous silver films using polystyrene templates. Nano-Micro Lett.

[34] Yan X, Yao J, Lu G, Li X, Zhang J, Han K (2005). Fabrication of non-close-packed arrays of colloidal spheres by soft lithography. Am Chem Soc.

[35] Lassiter JB, Knight MW, Mirin NA, Halas NJ (2009). Reshaping the plasmonic properties of an individual nanoparticle. Nano Lett.

